# Biotechnological Application of *Saccharomyces cerevisiae* and *Lactobacillus delbrueckii* sp. *bulgaricus* for Protein Enrichment of Fermented Unmalted and Malted Sorghum (*Sorghum bicolor* (L.) Moench)

**DOI:** 10.1155/2022/2264993

**Published:** 2022-02-03

**Authors:** Levi Yafetto, Christiana Naa Atsreh Nsiah-Asamoah, Emmanuel Birikorang, George Tawia Odamtten

**Affiliations:** ^1^Department of Molecular Biology and Biotechnology, School of Biological Sciences, College of Agriculture and Natural Sciences, University of Cape Coast, Cape Coast, Ghana; ^2^Department of Clinical Nutrition and Dietetics, School of Allied Health Sciences, College of Health and Allied Sciences, University of Cape Coast, Cape Coast, Ghana; ^3^Department of Laboratory Technology, School of Physical Sciences, College of Agriculture and Natural Sciences, University of Cape Coast, Cape Coast, Ghana; ^4^Department of Plant and Environmental Biology, School of Biological Sciences, College of Basic and Applied Sciences, University of Ghana, Legon, Accra, Ghana

## Abstract

This study evaluated changes in protein contents of malted and unmalted sorghum, and their formulated blends, after fermentation for 10 days at 25°C with mono and cocultures of *Saccharomyces cerevisiae* and *Lactobacillus delbrueckii* sp. *bulgaricus*. Fermentation of unmalted and malted sorghum and their formulated blends of 1 : 1 (*w*/*w*), 3 : 1 (*w*/*w*), and 1 : 3 (*w*/*w*) by *S*. *cerevisiae* and *L*. *bulgaricus* could increase their protein contents. Thus, there was an increase in protein content of fermented, malted sorghum by 68.40% for *S*. *cerevisiae*, 34.98% for *L*. *bulgaricus*, and 76.59% for cocultures of *S*. *cerevisiae* and *L*. *bulgaricus*; protein contents of fermented, unmalted sorghum also increased by 58.20, 39.36, and 55.00% for monoculture of *S*. *cerevisiae*, monoculture of *L*. *bulgaricus*, and coculture of *S*. *cerevisiae* and *L*. *bulgaricus*, respectively. *S*. *cerevisiae* was more effective in enriching protein content of the 1 : 3 (*w*/*w*) formulated blend of unmalted-malted sorghum by 77.59%; *L*. *bulgaricus* was more effective in enriching protein content of the 3 : 1 (*w*/*w*) unmalted-malted sorghum blend by 60.00%; coculture of *S*. *cerevisiae* and *L*. *bulgaricus* enriched the protein content of 3 : 1 (*w*/*w*) unmalted-malted sorghum substrate by 44.54%. Significant (*p* ≤ 0.05) increases in fat with corresponding decreases in carbohydrate and fibre contents were consistently recorded in malted and unmalted sorghum. In the formulated blends of sorghum, fat, carbohydrate, and fibre contents either increased or decreased erratically after fermentation. There were significantly (*p* ≤ 0.05) higher protein contents in malted sorghum, compared to unmalted sorghum. These findings show that solid-state microbial fermentation technology, using *S*. *cerevisiae* and *L*. *bulgaricus*, either as mono- or coculture, could effectively enrich the protein contents of unmalted and malted sorghum and their formulated blends. The implications of the findings for infant and adult nutrition are discussed, and future work to augment findings is suggested.

## 1. Introduction

The use of fermented food products predates the Biblical era, and it has since remained a tradition in many indigenous communities in Africa, Asia, Europe, and the Americas [1–3]. Thus, numerous fermented foods, for centuries and, perhaps, for millennia, have remained a central part of most cuisines in sub-Saharan Africa [4, 5]. The popularity of fermented foods in Africa is exemplified by the following foods and their countries of origin: *dawadawa* (Burkina Faso, Ghana, Nigeria, and Togo), *dégué*, (Burkina Faso), *ergo* (Ethiopia), *jben*, (Morocco), *kivunde* (Tanzania), *kule naoto* (Kenya), *poto poto* (Republic of Congo), *rigouta* (Tunisia), and *ugba* and *okpehe* (Nigeria) [6]. In Ghana, fermentation of cassava, corn, millet, sorghum, wheat, etc. is traditionally employed to produce locally fermented foods such as *banku* (a formulated blend of fermented cassava and corn dough), bread (fermented wheat dough), *gari* (fermented, roasted cassava grits), *kenkey* (fermented corn dough), *koko* (fermented corn porridge), *fura* (fermented millet dough), *kokonte* (fermented, dried cassava flour), *wagashi* (fermented cow milk to produce a traditional West African cottage cheese), and *pito* and *brukutu* (fermented African beer from sorghum). Notwithstanding the popularity of these fermented foods in Africa, there is a concern about the decline in their consumption, especially in urban areas. A comparative study conducted in rural and urban Kenya on the consumption patterns of fermented foods indicated that 83% of rural mothers reported that their families regularly consumed fermented foods and 66% confirmed that they fed their children with fermented foods; this is in contrast to urban settings, where 56% of mothers confirmed regular consumption of fermented foods by their families and 40% intimated that they fed their children with fermented foods [7]. The declining rate at which fermented foods are consumed in most communities could be attributed to two factors: (i) the misconception that fermented foods were bad and unhealthy and (ii) the substitution of traditional fermented foods mainly by some “westernized” commercial products such as cookies, soft drinks, and ready-to-eat canned foods—soups, sauces, fish, meat, mashed vegetables, etc. [6].

Sorghum (*Sorghum bicolor* (L.) Moench), a drought-resistant cereal crop, originally from Africa, is the fifth most important cereal produced in the world after maize, barley, wheat, and rice, and it is the third most cultivated cereal grain in Africa, after maize and rice [8–11]. It is a staple food in Africa and Asia (particularly, China and India), but it is used as livestock feed mostly in Australia and North and South America [12]. Thus, sorghum remains one of the most important cereal crops for multipurpose human utilization [9, 11, 13, 14], even though it is an underrated, nutrient-rich grain. Sorghum is enriched with important nutrients including carbohydrates, vitamins, and minerals; it serves as the main meal for millions of people around the globe, particularly in Africa [15, 16]. In addition, it has high antioxidant content (flavonoids, phenolics, terpenoids, etc.); consuming a diet rich in antioxidants can lower stress and inflammation in the body; a diet of sorghum provides 20% of the recommended fibre intake, promotes health, stabilizes blood sugar levels, and aids weight management [17]. The sorghum grain is a great source of protein and is gluten-free [15]. There are also results to show that certain varieties of *Sorghum bicolor* may affect critical biological processes that are important in diabetes and insulin resistance because of its phenolic content and high antioxidant properties that inhibit glycation [18]. However, one major drawback of sorghum is its poor starch and protein digestibility, which reduces its nutritional quality as compared to maize, wheat, or rice [19]. This drawback remains a major constraint to nutrition in infants and young children by limiting its usage in the preparation of complementary (weaning) foods. And so, to improve its values—nutritional qualities, palatability, and consumer appeal—for the market, sorghum ought to be augmented with food processing techniques [20–23]. It has been suggested that processing methods that expose the starch granules and protein matrix to digestion may help tackle the challenge of poor digestibility of sorghum [19]. One such food processing technique to make sorghum digestible is fermentation. Fermented food products, which are good sources of important bioactive compounds, are characterized by therapeutic, antimicrobial, antioxidant, probiotic, organoleptic, and cholesterol-lowering attributes [23–28].

Fermentation of solid substrates, also called solid-state fermentation, is a microbial process that involves the cultivation of microorganisms on solid substrates in the near absence of water or free-flowing water, where the substrate serves as a carbon and energy source [29]. The conventional approach of using solid-state fermentation with microbes, particularly the yeast, *Saccharomyces cerevisiae*, and the lactic acid bacteria (LAB), *Lactobacillus delbrueckii* sp. *bulgaricus*, which are generally regarded as safe (GRAS), presents an excellent biotechnological strategy for bioconversion of sorghum and other cereals into nutrient-rich fermented food rations for malnourished children in deprived communities in Ghana, as well as diabetics, pregnant women, vegetarians, and other convalescent adults with specialized nutritional needs. This approach is essential because *S*. *cerevisiae* and *L*. *bulgaricus* are conventionally used in bread making and yoghurt production, respectively, and are (i) good sources of enzymes, vitamins, and antioxidants and (ii) safe to consume [23–28]. *S*. *cerevisiae* (baker's yeast or “the yeast”) is the common yeast species in bread and sourdough. It has been used as a starter culture since the 19^th^ century [30], and its presence, for example, is known to affect aromatic potential in the fermentation of raw cocoa and its sensory attributes of chocolate [31]. It is the best-studied and one of the most widely used eukaryotes in a wide variety of industrial processes such as the production of wines, foods, and ethanol. [30]. *S*. *cerevisiae* has been used by Ariyajaroewong et al. [32] in repeated-batch ethanol production from sweet sorghum juice. Lactic acid bacteria, on the other hand, are bacteria (*Lactobacillus*, *Streptococcus*, *Enterococcus*, *Lactococcus*, *Bifidobacterium*, and *Leuconostoc*) that produce lactic acid as their major fermentation product [33]. The largest genus, *Lactobacillus*, contains about 80 species and is used in the production of fermented products that include pickle, sauerkraut, beer, wine, juices, cheese, yoghurt, and sausage [34, 35]. LAB fermentation is applied in the preparation of traditional foods in Africa; some of its benefits include increase in palatability of foods and availability of proteins and minerals, improvement in the preservative and detoxifying effects on food, and boost in the immune system that facilitates the body's fight against pathogenic bacterial infections [33].

There have been efforts by researchers to use microbial biotechnology to mitigate postharvest losses in cereal grains and to provide simple, efficiently produced, low-cost food rations during times of famine and, especially, to tackle hunger and malnutrition among children in deprived communities in developing countries, not excepting Ghana. Despite their potential and importance in enhancing the nutritional value of locally fermented foods, the prospect of using *S*. *cerevisiae* and *L*. *bulgaricus* for enriching the protein contents of sorghum is not yet well understood. Moreover, the use of *S*. *cerevisiae* and *L*. *bulgaricus* through solid-state fermentation of malted and unmated sorghum to increase protein content and other attributes has not been tried in Ghana. Thus, there is a paucity of information on the potential application of microbial biotechnology in augmenting the protein contents of most cereals in Ghana. Although fermentation of some food items for improved protein and nutritional attributes has been carried out using *S*. *cerevisiae* and *L*. *bulgaricus*, there is hardly any report in the pertinent literature on their use on sorghum grains for such purposes in Ghana. The objective of this paper was, therefore, to explore how microbial biotechnology can be utilized to improve protein content and other attributes of malted and unmalted grains of brown variety of sorghum (*Sorghum bicolor* (L.) Moench) by solid-state fermentation, using *S*. *cerevisiae* and *L*. *bulgaricus* as test microorganisms. The implications of the findings for infant and adult nutrition are discussed, and future work to augment findings is suggested.

## 2. Materials and Methods

### 2.1. *Sorghum bicolor* Grains

Brown variety of *Sorghum bicolor* grains was obtained from a local market in Cape Coast, Ghana.

### 2.2. *Saccharomyces cerevisiae* and *Lactobacillus delbrueckii* sp. *bulgaricus*

Commercially dried, instant baker's yeast, *Saccharomyces cerevisiae* (S. I. Lesaffre, France), was purchased from a supermarket in Cape Coast, Ghana, and stored in the laboratory at room temperature until ready to use. *Lactobacillus delbrueckii* sp. *bulgaricus* was isolated from a starter culture obtained from the Department of Molecular Biology and Biotechnology, University of Cape Coast, Ghana. Pure culture of the *L*. *bulgaricus* was maintained on de Man, Rogosa, and Sharpe (MRS) agar slants at 4°C and subcultured on MRS agar medium in Petri dishes every fortnight. It was used when needed.

### 2.3. de Man, Rogosa, and Sharpe Agar Medium

Sixty-two grams of powdered de Man, Rogosa, and Sharpe agar medium (Oxoid Ltd., England) was suspended in 1 liter of distilled water and gently heated to dissolve completely, after which it was dispensed in aliquots of 200 ml into five separate 500 ml Erlenmeyer flasks and sterilized by autoclaving at a pressure of 1.1 kg/cm^2^ at 121°C for 15 minutes; pH of the medium was adjusted to 6.2 ± 0.2 at 25°C and determined using a digital pH meter (EUTECH Instruments PC 700, India). The MRS agar medium was stored and used when needed.

### 2.4. de Man, Rogosa, and Sharpe Broth Medium

Fifty-two grams of MRS broth powder (Oxoid Ltd., England) was added to 1 liter of distilled water at 60°C and boiled to dissolve completely. The MRS broth medium obtained was dispensed in aliquots of 200 ml into five separate 500 ml Erlenmeyer flasks and sterilized by autoclaving at a pressure of 1.1 kg/cm^2^ at 121°C for 15 minutes; pH of the medium was adjusted to 6.2 ± 0.2 at 25°C. The MRS broth medium was stored and used when needed.

### 2.5. Yeast Extract-Peptone-Dextrose Agar and Broth Media

Yeast extract-peptone-dextrose (YPD) agar medium was prepared by dissolving 10 g of yeast extract, 20 g peptone, 20 g dextrose, and 15 g agar in 1 L distilled water. The YPD broth medium was prepared with the same quantities of yeast extract, peptone, and dextrose, without agar. The agar and broth media were sterilized by autoclaving at a pressure of 1.1 kg/cm^2^ at 121°C for 15 minutes; the pH of the media was adjusted to 6.5 ± 0.2 at 25°C, after which they were stored and used when needed.

### 2.6. Inoculum Suspension of *Lactobacillus bulgaricus*

Four MRS agar discs (5 mm in diameter) with *L*. *bulgaricus* were placed in 250 ml of sterilized MRS broth medium and gently shaken to dislodge bacterial cells from the discs to obtain *L*. *bulgaricus* inoculum suspension. The inoculum suspension was then placed on an electronic orbital shaker (IKA KS 260, Germany) and shaken at a speed of 150 rpm for 24 hours to ensure uniform proliferation and distribution of the bacterium in the MRS broth medium. The inoculum suspension of *L*. *bulgaricus* was used to inoculate sorghum substrates for fermentation studies.

### 2.7. Inoculum Suspension of *Saccharomyces cerevisiae*

Approximately 10 grams of *Saccharomyces cerevisiae* was added to a sterilized 1 L YPD broth medium and gently shaken to dislodge yeast cells to obtain uniform inoculum suspension of *S*. *cerevisiae*. The inoculum suspension of *S*. *cerevisiae* was then placed on an electronic orbit shaker and shaken at a speed of 150 rpm for 24 hours to ensure uniform proliferation and distribution of the yeast cells in the YPD broth medium. The inoculum suspension of *S*. *cerevisiae* was used to inoculate sorghum substrates for fermentation studies.

### 2.8. Unmalted and Malted Sorghum Substrates

**Figure 1:**
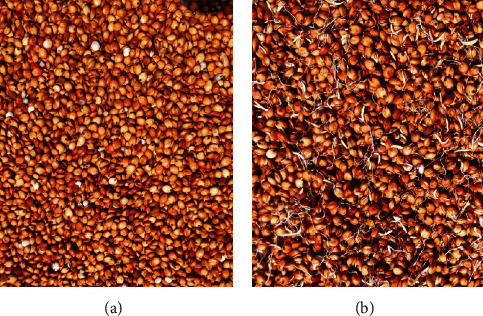
Unmalted (a) and malted (b) sorghum grains before milling for use in fermentation.

Sorghum grains were manually sorted and cleaned by handpicking chaff and debris, after which they were thoroughly washed twice under running tap water to ensure the grains were rid of any residual dirt. The sorghum grains (5 kg) were then seeped in 5% NaCl solution (5 L) for 4 hours to reduce microbial load and growth of resident microorganisms. The grains were rinsed under running water to get rid of residual NaCl solution and seeped in 5 L tap water for 24 hours in a clean 25 L  storage container. After seeping for 24 hours, the sorghum grains were drained and divided into two portions and separately processed to obtain *unmalted* and *malted* sorghum for fermentation studies as follows: (i) unmalted sorghum grains (Figure 1(a)) were obtained by sun drying soaked sorghum grains on wooden trays lined with muslin cloth for 3 days immediately after the 24-hour soaking period and (ii) malted sorghum grains (Figure 1(b)) were obtained by placing the soaked, wet sorghum grains under a humid ambient condition in a clean storage container for 2 days to allow them to germinate (sprout), after which they were sun-dried on wooden trays lined with muslin cloth for 3 days to halt further sprouting. The unmalted and malted sorghum grains were subsequently dried in an electric oven (Binder Model ED 23, Germany) at 50°C for 16 hours. The dried unmalted and malted sorghum grains were milled, sieved to obtain a fine powder using 3.35–4.00 mm size sieve mesh, and kept in airtight 170 × 250 mm resealable Ziploc bags for further use.

### 2.9. Fermentation of Sterilized Sorghum Grain Substrates

Key experiments involved solid-state fermentation of unmalted and malted sorghum substrates and the corresponding unmalted-malted formulated blends in weight by weight (*w*/*w*) ratios of 1 : 1, 3 : 1, and 1 : 3 (Table 1). All the sorghum substrates were fermented with mono and cocultures of *S*. *cerevisiae* and *L*. *bulgaricus* (Table 1).

**Figure 2:**
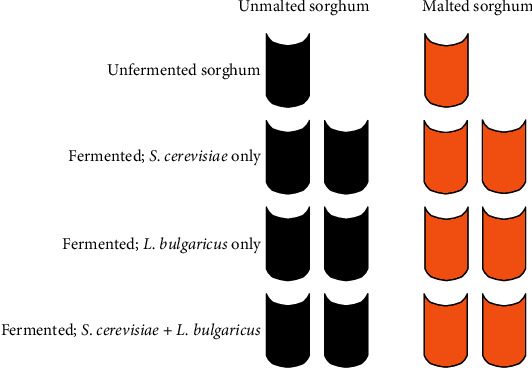
Process flow diagram of the solid-state fermentation method of unmalted sorghum (black vessels) and malted sorghum (orange vessels) with monocultures of *S*. *cerevisiae* and *L*. *bulgaricus* and coculture of *S*. *cerevisiae* and *L*. *bulgaricus*. Initial nitrogen (% N_2_) contents of unfermented unmalted and malted sorghum substrates, not inoculated with either *S*. *cerevisiae* or *L*. *bulgaricus* (controls), were determined at day 0 by the Kjeldahl method.

Unmalted and malted substrates were dispensed separately into seven 250 ml Erlenmeyer flasks, sterilized by autoclaving at a pressure of 1.1 kg/cm^2^ at 121°C for 15 minutes, and allowed to cool. Initial nitrogen (% N_2_) contents of 1 each of uninoculated unmalted and malted sorghum substrates (i.e., unfermented sorghum substrates) were determined by the Kjeldahl method. Subsequently, the remaining six sterilized unmalted and six sterilized malted sorghum substrates were treated as follows (Figure 2).

- Two 50 g unmalted sorghum and two 50 g malted sorghum substrates were separately inoculated with 40 ml each of inoculum suspension of *S*. *cerevisiae* only, as monocultures, at a moisture content of 50% (*w*/*v*) and fermented at 25°C
- Two 50 g unmalted sorghum and two 50 g malted sorghum substrates were separately inoculated with 40 ml each of inoculum suspension of *L*. *bulgaricus* only, as monoculture, at a moisture content of 50% (*w*/*v*) and fermented at 25°C
- Two 50 g unmalted sorghum and two 50 g malted sorghum substrates were separately inoculated with 20 ml each of inoculum suspensions of *S*. *cerevisiae* and *L*. *bulgaricus*, as cocultures, at a moisture content of 50% (*w*/*v*) and fermented at 25°C

% N_2_ of the one fermented unmalted and malted sorghum substrate under each treatment was determined in triplicate after 5 days of fermentation (Figure 2); subsequently, % N_2_ of the remaining fermented unmalted and malted sorghum substrates was determined after 10 days of fermentation (Figure 2). The procedure was repeated for the formulated blends of unmalted and malted sorghum substrates (Table 1).

### 2.10. Proximate Analyses of Unfermented and Fermented Sorghum Substrates

The % N_2_ of sorghum substrates determined initially at day 0 and after 5 and 10 days of fermentation was used to calculate crude percentage protein using the formula %N_2_ × 6.25, where 6.25 is the protein conversion factor. A percentage increase in protein contents of the fermented sorghum was subsequently calculated according to the formula used by Yafetto et al. [36]. All procedures for proximate analyses for ash, carbohydrate, crude fat, crude fibre, and moisture contents of unfermented and fermented sorghum substrates were conducted in triplicate following AOAC methods [37]. The respective percentage crude ash, fat, fibre, and carbohydrate were calculated as follows:

(1) $$\begin{array}{c} \begin{array}{c} \text{Crude ash }\left(\%\right)=\left[\text{Weight of ash }\left(\text{g}\right)÷\text{Weight of sample }\left(\text{g}\right)\right]\times 100,\\ \text{Crude fat }\left(\%\right)=\left[\text{Weight of oil }\left(\text{g}\right)÷\text{Weight of sample }\left(\text{g}\right)\right]\times 100,\\ \text{Crude fibre }\left(\%\right)=\left[\text{Weight loss through ashing }\left(\text{g}\right)÷\text{Weight of sample }\left(\text{g}\right)\right]\times 100,\\ \text{Carbohydrate }\left(\%\right)=\left[100\%-\left(\%\text{moisture}+\%\text{crude protein}+\%\text{crude fat}+\%\text{crude fibre}+\%\text{crude ash}\right)\right]. \end{array} \end{array}$$

### 2.11. Data Analysis

One-Way Analysis of Variance (ANOVA) was performed on the data using the Statistical Package for the Social Sciences (SPSS) version 25.0 (IMB SPSS Statistics, USA). Means were separated using the Tukey post hoc test at a 95% confidence level (*p* ≤ 0.05). The final results were expressed as means ± standard deviation (SD).

## 3. Results

### 3.1. Protein Enrichment and Proximate Composition of Fermented Unmalted Sorghum

**Figure 3:**
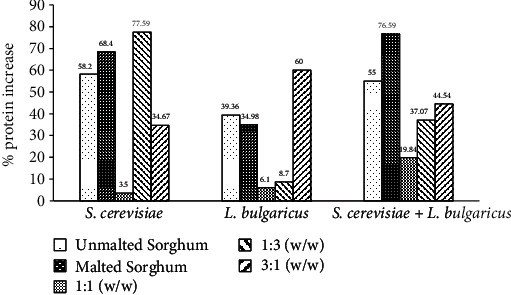
Percentage (%) increase in protein contents of malted and unmalted sorghum and their formulated blends after 10 days of fermentation.

The initial protein content (%) of unmalted sorghum at day 0 was 13.11 ± 0.21 (Table 2). The protein contents of the unmalted sorghum substrates inoculated with *S*. *cerevisiae*, *L*. *bulgaricus*, and coculture of *S*. *cerevisiae* and *L*. *bulgaricus* showed a significantly (*p* ≤ 0.05) steady increase at 20.74 ± 0.12, 18.27 ± 0.20, and 20.32 ± 0.14, respectively, after 10 days of fermentation (Table 2). Consequently, the percentage increase in protein content of the unmalted sorghum was 58.20% for *S*. *cerevisiae*, 39.36% for *L*. *bulgaricus*, and 55.00% for coculture of *S*. *cerevisiae* and *L*. *bulgaricus* (Figure 3). There was a significant (*p* ≤ 0.05) increase in fat content in all unmalted sorghum substrates fermented with *S*. *cerevisiae*, *L*. *bulgaricus*, and coculture of *S*. *cerevisiae* and *L*. *bulgaricus* at 3.84 ± 0.21, 3.78 ± 0.20, and 3.85 ± 0.30, respectively (Table 2). Interestingly, there were remarkable significant (*p* ≤ 0.05) decreases in both carbohydrate and fibre contents in all unmalted sorghum substrates fermented with *S*. *cerevisiae*, *L*. *bulgaricus*, and coculture of *S*. *cerevisiae* and *L*. *bulgaricus* (Table 2). Whereas there was a significant (*p* ≤ 0.05) increase in the ash content of unmalted sorghum fermented with *S*. *cerevisiae* at 1.55 ± 0.03, there were an insignificant (*p* ≤ 0.05) increase in ash content in unmalted sorghum fermented with *L*. *bulgaricus* at 1.54 ± 0.02 and an insignificant (*p* ≤ 0.05) decrease in unmalted sorghum after 10 days of fermentation with coculture of *S*. *cerevisiae* and *L*. *bulgaricus* at 1.47 ± 0.02 (Table 2).

### 3.2. Protein Enrichment and Proximate Composition of Fermented Malted Sorghum

The initial protein content (%) of malted sorghum at day 0 was 12.69 ± 0.04 (Table 3). There was a significant (*p* ≤ 0.05) steady increase in the protein contents of malted sorghum substrates inoculated with *S*. *cerevisiae* and coculture of *S*. *cerevisiae* and *L*. *bulgaricus* at 21.37 ± 0.14 and 22.41 ± 0.40, respectively, after 10 days of fermentation (Table 3). However, malted sorghum inoculated with *L*. *bulgaricus* had an initial increase in protein content at day 5 (19.52 ± 0.40), after which there was a decrease in its protein content at 17.13 ± 0.20 after 10 days of fermentation (Table 3). Consequently, the percentage increase in protein contents of the malted sorghum substrates was 68.40, 34.98, and 76.59% for monoculture of *S*. *cerevisiae*, monoculture of *L*. *bulgaricus*, and coculture of *S*. *cerevisiae* and *L*. *bulgaricus*, respectively (Figure 3). There was a significant (*p* ≤ 0.05) increase in fat content in all malted sorghum substrates fermented with *S*. *cerevisiae*, *L*. *bulgaricus*, and coculture of *S*. *cerevisiae* and *L*. *bulgaricus* at 4.01 ± 0.04, 3.27 ± 0.10, and 3.95 ± 0.21, respectively (Table 3). However, like fermented unmalted sorghum, there were significant decreases (*p* ≤ 0.05) in carbohydrate and fibre contents in all malted sorghum substrates fermented with *S*. *cerevisiae*, *L*. *bulgaricus*, and coculture of *S*. *cerevisiae* and *L*. *bulgaricus* (Table 3). Interestingly, there was no significant increase in the ash content of malted sorghum fermented with *S*. *cerevisiae* (1.65 ± 0.01), whereas there were significant (*p* ≤ 0.05) decreases in ash content in malted sorghum fermented with *L*. *bulgaricus* at 1.35 ± 0.02 and with coculture of *S*. *cerevisiae* and *L*. *bulgaricus* at 1.45 ± 0.02 (Table 3).

### 3.3. Protein Enrichment and Proximate Composition of Fermented Formulated Blends of Sorghum

In the present study, the initial protein (%) contents of fermented unmalted-malted formulated blends of sorghum substrates with ratios of 1 : 1 (*w*/*w*), 1 : 3 (*w*/*w*), and 3 : 1 (*w*/*w*) were 16.19 ± 0.14, 13.57 ± 0.14, and 12.75 ± 0.17, respectively (Tables 4–6). Protein contents of all fermented blends increased significantly (*p* ≤ 0.05) with *S*. *cerevisiae*, *L*. *bulgaricus*, and cocultures of *S*. *cerevisiae* and *L*. *bulgaricus*, except the 1 : 1 (*w*/*w*) unmalted-malted formulated blend fermented with coculture of *S*. *cerevisiae* and *L*. *bulgaricus*, where there was an insignificant increase in protein content at 19.51 ± 0.42 after 10 days of fermentation (Tables 4–6). There was an overall percentage increase in the protein contents of all the formulated blends after 10 days of fermentation: the highest percentage increase of 77.59% was determined with *S*. *cerevisiae*, followed by a percentage increase of 60.00 and 44.54% in the 3 : 1 (*w*/*w*) blend fermented with *L*. *bulgaricus* and coculture of *S*. *cerevisiae* and *L*. *bulgaricus*, respectively (Figure 3).

There was a significant (*p* ≤ 0.05) decrease in fat contents of the 1 : 1 (*w*/*w*) unmalted-malted blend fermented with *S*. *cerevisiae* (2.31 ± 0.11) and *L*. *bulgaricus* (2.31 ± 0.08), with an insignificant decrease at 2.58 ± 0.07 after fermentation with coculture of *S*. *cerevisiae* and *L*. *bulgaricus* (Table 4). Carbohydrate contents of a 1 : 1 (*w*/*w*) formulated blend of sorghum substrates fermented with *S*. *cerevisiae* increased insignificantly at 72.69 ± 0.15, while the carbohydrate content insignificantly decreased with *L*. *bulgaricus* and coculture of *S*. *cerevisiae* and *L. bulgaricus* at 72.35 ± 0.05 and 70.93 ± 0.31, respectively (Table 4). Fibre contents in a 1 : 1 (*w*/*w*) formulated blend of sorghum increased insignificantly with *S*. *cerevisiae* but increased (*p* ≤ 0.05) significantly with *L*. *bulgaricus*; however, the fibre content decreased (*p* ≤ 0.05) significantly when fermented with coculture of *S*. *cerevisiae* and *L*. *bulgaricus* (Table 4). The ash content decreased (*p* ≤ 0.05) significantly from 1.61 ± 0.07 to 1.27 ± 0.02, 1.18 ± 0.02, and 1.41 ± 0.01 when the 1 : 1 (*w*/*w*) blend was fermented with *S*. *cerevisiae*, *L*. *bulgaricus*, and coculture of *S*. *cerevisiae* and *L*. *bulgaricus*, respectively (Table 4).

Insignificant decreases in fat contents were reported for the 1 : 3 (*w*/*w*) sorghum blend fermented with *S*. *cerevisiae*, *L*. *bulgaricus*, and coculture of *S*. *cerevisiae* and *L*. *bulgaricus*; on the contrary, there was a significant decline in ash contents of samples fermented with *S*. *cerevisiae*, *L*. *bulgaricus*, and coculture of *S*. *cerevisiae* and *L*. *bulgaricus* (Table 5). Carbohydrate contents of the 1 : 3 (*w*/*w*) formulated blend of sorghum fermented with *S*. *cerevisiae*, *L*. *bulgaricus*, and coculture of *S*. *cerevisiae* and *L*. *bulgaricus* decreased (*p* ≤ 0.05) significantly to 64.52 ± 0.24, 74.31 ± 0.14, and 70.43 ± 0.20, respectively, from an initial carbohydrate content of 74.97 ± 0.13 (Table 5). Interestingly, the fibre content of the blend, after 10 days of fermentation, marginally increased from an initial 6.89 ± 0.10 to 6.99 ± 00.6, 6.92 ± 0.07, and 6.91 ± 0.02, respectively, for *S*. *cerevisiae*, *L*. *bulgaricus*, and coculture of *S*. *cerevisiae* and *L*. *bulgaricus* (Table 5).

There was a marginal decline in fat contents from 2.99 ± 0.10 to 2.75 ± 0.05 and 2.93 ± 0.14 in the 3 : 1 (*w*/*w*) sorghum blend fermented with *S*. *cerevisiae* and coculture of *S*. *cerevisiae* and *L*. *bulgaricus*, respectively, whereas there was an insignificant increase in fat content with *L*. *bulgaricus* at 3.00 ± 0.08 (Table 6). Carbohydrate contents of the 3 : 1 (*w*/*w*) sorghum blend fermented with *S*. *cerevisiae*, *L. bulgaricus*, and coculture of *S. cerevisiae* and *L. bulgaricus* decreased (*p* ≤ 0.05) significantly to 71.81 ± 0.05, 68.61 ± 0.10, and 70.44 ± 0.54, respectively, from an initial carbohydrate content of 75.63 ± 0.21 (Table 6). Similarly, there was a significant (*p* ≤ 0.05) decrease in fibre contents of the 3 : 1 (*w*/*w*) blend fermented with *L*. *bulgaricus* (at 6.73 ± 0.06) and coculture of *S*. *cerevisiae* and *L*. *bulgaricus* (6.88 ± 0.07), except the sorghum blend fermented with *S*. *cerevisiae*, where an increase in the fibre content was not significant (Table 6).

## 4. Discussion

The key fermenters of foods in many parts of the world are *Bacillus*, *Lactobacillus*, *Streptococcus* spp., and other filamentous fungi, to mention a few. According to some workers [4, 6, 27, 38–40], most popular indigenous cuisines in sub-Saharan Africa are mainly locally fermented foods. Microorganisms that play major roles in the fermentation of food have probiotic, antioxidant, organoleptic, and antimicrobial activities [6]. This present study was carried out to assess the use of microbial biotechnology by *in vitro* solid-state fermentation for the enrichment of protein contents of malted and unmalted sorghum grains in different proportions.

Results show that these formulated sorghum substrates (malted and unmalted) inoculated with single or cocultures of *S*. *cerevisiae* and *L*. *bulgaricus* increased protein content in various blends varying from 6.10 to 77.59% (Figure 3, Tables 4–6). Yagoub et al. [41] reported a protein content increase in the range of 9.13–12.7% for two local varieties of Sudanese fermented sorghum (*S*. *bicolor*) using LAB strains. Afify et al. [42] also found increases in protein content in fermented sorghum of different varieties, not excepting [43] who also reported increases in sorghum fermented with *S*. *cerevisiae*. Interestingly, compared to all the above-cited instances, where increases in protein contents were recorded, our present study shows much higher protein content, except for the 1 : 1 and 3 : 1 (*w*/*w*) of unmalted-malted formulated blends of sorghum, where 3.50 and 6.10% increases were, respectively, obtained in 10 days (Figure 3; Tables 2–6). This is not surprising because of presumable varietal differences in the sorghum grains used in each instance, as well as changes in cultivation conditions under which these studies were carried out. For example, differences in temperature, moisture contents, pH, inoculation size of test microorganisms, and duration of fermentation period could influence, to different extents, the protein content and other nutritional composition of fermented grains and cereals. Unlike other studies, the coinoculation of sorghum substrates with *S*. *cerevisiae* and *L*. *bulgaricus*, in our study, may also have an added advantage in the sense that lactic acid produced by *L*. *bulgaricus* may provide a more acidic substrate for *S*. *cerevisiae* to effectively utilize. Therefore, we conjecture that the relatively higher protein content obtained in our studies using malted and unmalted sorghum may be attributed to the ability of both *S*. *cerevisiae* and *L*. *bulgaricus* to produce a miscellany of enzymes that could break down the substrates into composite amino acids and other metabolic products to be used by the fermenting organisms for their growth [44–48]. Future studies should profile the amino acid contents of fermented cereals and grains used in the preparation of African meals.

There was another added effect that could have contributed to the enhanced protein content in this present study: the traditional malting process was combined with fermentation. The tradition of combined processes of malting and fermentation in the preparation of African foods has been reported to enrich the nutritional content of sorghum and other grains and cereals [19]. *In vitro* studies with sorghum have demonstrated that malting promotes the production of hydrolytic enzymes such as amylases, proteases, and phytases, which are absent in nongerminating grains [19]. The malting process improves also protein digestibility and other processing characteristics, increases vitamin C content and availability of mineral elements such as Fe, Zn, and P, enhances the synthesis of amino acids, and even improves flavour and aesthetic colour of the substrate [49, 50]. Fermentation, on the other hand, utilizes carbohydrates as substrates by employing enzymatic activities of yeasts, filamentous fungi, or bacteria. The fermentation process enhances also the organoleptic properties (flavour, texture, taste, palatability, appearance, etc.), increases the profile of vitamins, minerals, and amino acids, and improves bioavailability and digestibility of the substrate. Furthermore, the fermentation process can remove antinutritional factors (alkaloids, flavonoids, tannins, oxalates, etc.) from a substrate [38, 45]. Future studies that apply both malting and fermentation to augment nutritional qualities of sorghum and other grains on a large scale in fermentors should be considered.

The fibre content of unmalted and malted sorghum in this study decreased significantly (*p* ≤ 0.05) after 10 days of fermentation after inoculation with single or mixed cultures of *S*. *cerevisiae* and *L*. *bulgaricus* (Tables 2 and 3). In contrast, the fibre content of fermented blends of unmalted-malted sorghum did not follow the same trend and either increased, decreased, or remained unaffected (Tables 4–6). The present data cannot explain this trend. However, results from other investigators showed increases in fibre content in malted, fermented sorghum in contrast to our data [51, 52]. It is conjectured that cultural conditions and varietal differences in the sorghum used may partly explain these contrasting results. Interestingly, [38, 47, 53, 54] using different substrates including soybeans found a decrease in fibre content as a result of fermentation. The general decrease in the fibre content of substrate in this present study suggests the probable ability of *S*. *cerevisiae* and *L*. *bulgaricus* to metabolize fibre components through induced enzymatic breakdown during the fermentation process in the unmalted and malted substrates as compared to the formulated blends of fermented unmalted-malted sorghum.

Fat content increased in unmalted and malted sorghum during fermentation (Tables 2 and 3) in contrast with the formulated blends of unmalted-malted sorghum, where fat content decreased significantly (*p* ≤ 0.05). This increase in fat during fermentation may be due to a change in the metabolic pathway from glycolytic to fat metabolic cycle by utilizing carbohydrates as a substrate for fat formation. It was, therefore, not surprising that the carbohydrate content in all the sorghum substrates in this study decreased with an increasing period of fermentation presumably due to the hydrolysis of carbohydrates into simple compounds which served as precursors for fat formation. Indeed, Afify et al. [42] have previously reported an increase in fat content to about 3.58–3.91% in three white varieties of sorghum. On the other hand, the reported decrease in fat content in the formulated blends of unmalted-malted sorghum in the present study has also been reported by ElMaki et al. [55] who used a different variety of sorghum. This decrease in fat content may be attributed to increased lipase activity, where the first step in such utilization of fat is by its hydrolysis to glycerol and fatty acids. Future studies could ascertain this viewpoint. In such future studies, lipase activity will be determined as it can be tested in a water-soluble synthetic substrate. The production of aromatic compounds through the breakdown of fatty acids and glycerol during fermentation has also been shown by [46, 47, 56]. If indeed, rancidity in fat during storage is caused by higher fat content, affecting flavour in the process [57], then fermentation with LAB strains could serve as a useful purpose in reducing the high-fat content of sorghum [10, 58], as was found in the formulated blend (unmalted-malted sorghum substrate) inoculated with either monocultures of *S*. *cerevisiae* and *L*. *bulgaricus* or both (Tables 4–6). High levels of polyunsaturated fat in sorghum after malting may improve its health benefits in reducing cholesterol levels in consumer foods for people with the propensity for hypertension and cardiovascular diseases. Furthermore, enzymatic action by fermentation microbes may degrade and, in the process, decrease antinutritional factors and assist in the breakdown of complex macromolecules into simple, more digestible forms [59].

The ash content reported in this present study changed inconsistently depending on the sorghum substrate used for the solid-state fermentation (Tables 2–6). These results are at variance with the findings of some workers [38, 48, 60] who reported an increase in ash content of maize, pigeon pea, sorghum, and soybean after fermentation with other LAB strains. Ash content of substrates is an indicator of the mineral components and content, and as such, a decline in the ash of sorghum substrate across the board may be partially attributed to a concomitant reduction in nutritional and mineral content of the formulated sorghum substrates before and after malting and fermentation. Studies in this regard are in progress and will be reported in a subsequent paper.

## 5. Conclusion

The data presented in this paper show the efficacy of *S*. *cerevisiae* and *L*. *bulgaricus*, inoculated singly or coinoculated, in the enrichment of the protein content of sorghum. This possibly implies that the fermentation process can be used to produce gruels for use as food rations for improved human nutrition and healthy well-being. There is a miscellany of interventions that could be useful for health improvement using plant-based, protein-rich sorghum for the production of formula foods for weaning children, production of nondairy probiotics and vegetarian food products, and a possible lowering of hyperlipidemia in cardiovascular diseases. A lot more preliminary clinical trials using human and animal models are required to evaluate the suitability of malting and fermentation in serving these purposes. This paper is only a springboard for future studies for wider benefits. Indeed, malting and fermentation are low-cost cottage processes in preparing most fermented foods in African households, and women in the rural and urban communities can be educated on the nutritional and health benefits of consuming hygienic products arising from this research.

## Figures and Tables

**Table 1:** Sorghum substrates and treatments employed in their fermentation.

Sorghum substrate	Treatment		
	*S*. *cerevisiae*	*L*. *bulgaricus*	*S*. *cerevisiae*+*L*. *bulgaricus*
50 g unmalted	√	√	√
50 g malted	√	√	√
1 : 1	√	√	√
1 : 3	√	√	√
3 : 1	√	√	√

**Table 2:** Changes in the chemical composition of fermented, unmalted sorghum with monocultures of *S*. *cerevisiae* or *L*. *bulgaricus* and a coculture of *S*. *cerevisiae* and *L*. *bulgaricus* (data presented are based on the dry matter). ^a–c^Means within a column with different superscripts are significantly different (*p* ≤ 0.05). Results expressed as mean (*n* = 3) ± SD (standard deviation).

Culture	Day	Dry matter	Moisture	Ash	Fat	Fibre	Carbohydrate	Protein
*S*. *cerevisiae*	0	91.24 ± 0.10^a^	8.76 ± 0.10^a^	1.51 ± 0.06^ab^	3.00 ± 0.07^a^	8.73 ± 0.14^a^	73.64 ± 0.45^a^	13.11 ± 0.21^a^
	5	49.42 ± 0.22^b^	50.58 ± 0.22^b^	1.34 ± 0.11^a^	3.90 ± 0.03^b^	8.62 ± 0.11^a^	67.27 ± 0.40^b^	18.87 ± 0.40^b^
	10	42.43 ± 0.44^c^	57.57 ± 0.44^c^	1.55 ± 0.03^b^	3.84 ± 0.21^b^	7.42 ± 0.20^b^	66.46 ± 0.20^b^	20.74 ± 0.12^c^
*L*. *bulgaricus*	0	91.24 ± 0.10^a^	8.76 ± 0.10^a^	1.51 ± 0.06^a^	3.00 ± 0.07^a^	8.73 ± 0.14^a^	73.64 ± 0.45^a^	13.11 ± 0.21^a^
	5	49.31 ± 0.41^b^	50.68 ± 0.41^b^	1.35 ± 0.06^b^	3.20 ± 0.07^a^	8.11 ± 0.07^b^	69.88 ± 0.30^b^	17.45 ± 0.34^b^
	10	43.61 ± 0.40^c^	56.39 ± 0.40^c^	1.54 ± 0.02^a^	3.78 ± 0.20^b^	7.07 ± 0.11^c^	69.34 ± 0.12^b^	18.27 ± 0.20^c^
*S*. *cerevisiae*+*L*. *bulgaricus*	0	91.24 ± 0.10^a^	8.76 ± 0.10^a^	1.51 ± 0.06^a^	3.00 ± 0.07^a^	8.73 ± 0.14^a^	73.64 ± 0.45^a^	13.11 ± 0.21^a^
	5	49.44 ± 0.63^b^	50.56 ± 0.63^b^	1.40 ± 0.10^a^	3.67 ± 0.06^b^	7.97 ± 0.12^b^	69.13 ± 0.22^b^	17.84 ± 0.30^b^
	10	42.69 ± 0.41^c^	57.31 ± 0.41^c^	1.47 ± 0.02^a^	3.85 ± 0.30^b^	7.04 ± 0.11^c^	67.32 ± 0.20^c^	20.32 ± 0.14^c^

**Table 3:** Changes in the chemical composition of fermented, malted sorghum with monocultures of *S*. *cerevisiae* or *L*. *bulgaricus* and a coculture of *S*. *cerevisiae* and *L*. *bulgaricus* (data presented are based on the dry matter). ^a–c^Means within a column with different superscripts are significantly different (*p* ≤ 0.05). Results expressed as mean (*n* = 3) ± SD (standard deviation).

Culture	Day	Dry matter	Moisture	Ash	Fat	Fibre	Carbohydrate	Protein
*S*. *cerevisiae*	0	91.71 ± 0.10^a^	8.34 ± 0.10^a^	1.62 ± 0.10^a^	2.88 ± 0.07^a^	8.49 ± 0.16^a^	74.31 ± 0.10^a^	12.69 ± 0.04^a^
	5	50.48 ± 0.50^b^	49.52 ± 0.50^b^	1.39 ± 0.10^b^	3.32 ± 0.10^b^	8.22 ± 0.04^a^	67.63 ± 0.80^b^	19.44 ± 0.70^b^
	10	43.06 ± 0.06^c^	56.94 ± 0.06^c^	1.65 ± 0.01^a^	4.01 ± 0.04^c^	7.41 ± 0.40^b^	65.56 ± 0.40^c^	21.37 ± 0.14^c^
*L*. *bulgaricus*	0	91.71 ± 0.10^a^	8.34 ± 0.10^a^	1.62 ± 0.10^a^	2.88 ± 0.07^a^	8.49 ± 0.16^a^	74.31 ± 0.10^a^	12.69 ± 0.04^a^
	5	49.22 ± 0.30^b^	50.78 ± 0.30^b^	1.24 ± 0.04^b^	3.11 ± 0.10^b^	7.82 ± 0.12^b^	68.31 ± 0.55^b^	19.52 ± 0.40^b^
	10	45.81 ± 0.13^c^	54.19 ± 0.13^c^	1.35 ± 0.02^b^	3.27 ± 0.10^b^	7.34 ± 0.23^c^	70.91 ± 0.40^c^	17.13 ± 0.20^c^
*S*. *cerevisiae*+*L*. *bulgaricus*	0	91.71 ± 0.10^a^	8.34 ± 0.10^a^	1.62 ± 0.10^a^	2.88 ± 0.07^a^	8.49 ± 0.16^a^	74.31 ± 0.10^a^	12.69 ± 0.04^a^
	5	49.46 ± 0.31^b^	50.54 ± 0.31^b^	1.37 ± 0.03^b^	3.28 ± 0.05^b^	7.56 ± 0.10^b^	69.61 ± 0.20^b^	18.17 ± 0.14^b^
	10	38.49 ± 0.30^c^	61.51 ± 0.30^c^	1.45 ± 0.02^b^	3.95 ± 0.21^c^	7.26 ± 0.12^b^	64.93 ± 0.10^c^	22.41 ± 0.40^c^

**Table 4:** Changes in the chemical composition of fermented, formulated blends of unmalted and malted sorghum in a ratio of 1 : 1 (*w*/*w*) with monocultures of *S*. *cerevisiae* or *L*. *bulgaricus* and a coculture of *S*. *cerevisiae* and *L*. *bulgaricus* (data presented are based on the dry matter). ^a–c^Means within a column with different superscripts are significantly different (*p* ≤ 0.05). Results expressed as mean (*n* = 3) ± SD (standard deviation).

Culture	Day	Dry matter	Moisture	Ash	Fat	Fibre	Carbohydrate	Protein
*S. cerevisiae*	0	89.10 ± 0.23^a^	10.90 ± 0.23^a^	1.61 ± 0.07^a^	2.97 ± 0.07^a^	6.80 ± 0.01^a^	72.44 ± 0.11^a^	16.19 ± 0.14^a^
	5	49.77 ± 0.04^b^	50.23 ± 0.04^b^	1.24 ± 0.04^b^	2.68 ± 0.05^b^	6.85 ± 0.06^a^	70.76 ± 0.07^b^	18.45 ± 0.07^b^
	10	50.29 ± 0.08^c^	49.71 ± 0.08^c^	1.27 ± 0.02^b^	2.31 ± 0.11^c^	6.88 ± 0.10^a^	72.69 ± 0.15^a^	16.85 ± 0.24^c^
*L. bulgaricus*	0	89.10 ± 0.23^a^	10.90 ± 0.23^a^	1.61 ± 0.07^a^	2.97 ± 0.07^a^	6.80 ± 0.01^a^	72.44 ± 0.11^a^	16.19 ± 0.14^a^
	5	49.27 ± 0.32^b^	50.73 ± 0.32^b^	1.14 ± 0.05^b^	2.89 ± 0.08^a^	6.99 ± 0.01^b^	72.44 ± 0.44^a^	16.54 ± 0.40^a^
	10	45.57 ± 0.43^c^	54.43 ± 0.43^c^	1.18 ± 0.02^b^	2.31 ± 0.08^b^	6.89 ± 0.04^c^	72.35 ± 0.05^a^	17.27 ± 0.08^b^
*S. cerevisiae+L. bulgaricus*	0	89.10 ± 0.23^a^	10.90 ± 0.23^a^	1.61 ± 0.07^a^	2.97 ± 0.07^ab^	6.80 ± 0.01^ab^	72.44 ± 0.11^a^	16.19 ± 0.14^a^
	5	50.47 ± 0.17^b^	49.96 ± 0.42^b^	1.32 ± 0.01^b^	2.72 ± 0.04^ab^	5.91 ± 0.05^b^	71.68 ± 0.24^b^	18.35 ± 0.23^b^
	10	42.53 ± 0.37^c^	57.47 ± 0.37^c^	1.41 ± 0.01^b^	2.58 ± 0.07^b^	5.56 ± 0.10^c^	70.93 ± 0.31^a^	19.51 ± 0.42^a^

**Table 5:** Changes in the chemical composition of fermented, formulated blends of unmalted and malted sorghum in a ratio of 1 : 3 (*w*/*w*) with monocultures of *S*. *cerevisiae* or *L*. *bulgaricus* and a coculture of *S*. *cerevisiae* and *L*. *bulgaricus* (data presented are based on the dry matter). ^a–c^Means within a column with different superscripts are significantly different (*p* ≤ 0.05). Results expressed as mean (*n* = 3) ± SD (standard deviation).

Culture	Day	Dry matter	Moisture	Ash	Fat	Fibre	Carbohydrate	Protein
*S. cerevisiae*	0	88.39 ± 0.21^a^	11.61 ± 0.21^a^	1.58 ± 0.03^a^	2.99 ± 0.03^a^	6.89 ± 0.10^a^	74.97 ± 0.13^a^	13.57 ± 0.14^a^
	5	49.30 ± 0.30^b^	50.70 ± 0.30^b^	1.17 ± 0.04^b^	2.25 ± 0.01^b^	7.05 ± 0.10^a^	74.99 ± 0.40^a^	14.54 ± 0.24^b^
	10	38.74 ± 0.21^c^	61.25 ± 0.21^c^	1.44 ± 0.02^c^	2.98 ± 0.06^a^	6.99 ± 0.06^a^	64.52 ± 0.24^b^	24.07 ± 0.17^c^
*L. bulgaricus*	0	88.39 ± 0.21^a^	11.61 ± 0.21^a^	1.58 ± 0.03^a^	2.99 ± 0.03^a^	6.89 ± 0.10^a^	74.97 ± 0.13^a^	13.57 ± 0.14^a^
	5	50.32 ± 0.70^b^	49.68 ± 0.70^b^	1.16 ± 0.50^b^	2.35 ± 0.07^b^	6.84 ± 0.05^a^	74.46 ± 0.18^b^	15.19 ± 0.10^b^
	10	49.67 ± 0.37^b^	50.33 ± 0.37^b^	1.17 ± 0.03^b^	2.85 ± 0.12^a^	6.92 ± 0.07^a^	74.31 ± 0.14^b^	14.75 ± 0.05^c^
*S. cerevisiae+L. bulgaricus*	0	88.39 ± 0.21^a^	11.61 ± 0.21^a^	1.58 ± 0.03^a^	2.99 ± 0.03^a^	6.89 ± 0.10^a^	74.97 ± 0.13^a^	13.57 ± 0.14^a^
	5	48.90 ± 0.17^b^	51.10 ± 0.17^b^	1.19 ± 0.02^b^	2.53 ± 0.40^a^	6.87 ± 0.10^a^	73.05 ± 0.40^b^	16.36 ± 0.10^b^
	10	43.71 ± 0.12^c^	56.29 ± 0.12^c^	1.24 ± 0.04^b^	2.81 ± 0.10^a^	6.91 ± 0.02^a^	70.43 ± 0.20^c^	18.60 ± 0.11^c^

**Table 6:** Changes in the chemical composition of fermented, formulated blends of unmalted and malted sorghum in a ratio of 3 : 1 (*w*/*w*) with monocultures of *S*. *cerevisiae* or *L*. *bulgaricus* and a coculture of *S*. *cerevisiae* and *L*. *bulgaricus* (data presented are based on the dry matter). ^a–c^Means within a column with different superscripts are significantly different (*p* ≤ 0.05). Results expressed as mean (*n* = 3) ± SD (standard deviation).

Culture	Day	Dry matter	Moisture	Ash	Fat	Fibre	Carbohydrate	Protein
*S. cerevisiae*	0	89.62 ± 0.12^a^	10.23 ± 0.12^a^	1.62 ± 0.07^a^	2.99 ± 0.10^a^	7.01 ± 0.02^a^	75.63 ± 0.21^a^	12.75 ± 0.17^a^
	5	50.81 ± 0.22^b^	49.19 ± 0.22^b^	1.24 ± 0.02^b^	2.74 ± 0.15^a^	6.67 ± 0.06^b^	71.15 ± 0.30^b^	18.19 ± 0.10^b^
	10	50.53 ± 0.15^b^	49.47 ± 0.15^b^	1.23 ± 0.01^b^	2.75 ± 0.05^a^	7.04 ± 0.05^a^	71.81 ± 0.05^c^	17.17 ± 0.02^c^
*L. bulgaricus*	0	89.62 ± 0.12^a^	10.23 ± 0.12^a^	1.62 ± 0.07^a^	2.99 ± 0.10^a^	7.01 ± 0.02^a^	75.63 ± 0.21^a^	12.75 ± 0.17^a^
	5	47.30 ± 0.14^b^	52.70 ± 0.14^b^	1.08 ± 0.10^b^	2.89 ± 0.04^a^	6.85 ± 0.05^b^	72.26 ± 0.05^b^	16.92 ± 0.05^b^
	10	45.23 ± 0.27^c^	54.77 ± 0.27^c^	1.25 ± 0.01^c^	3.00 ± 0.08^a^	6.73 ± 0.06^b^	68.61 ± 0.10^c^	20.40 ± 0.22^c^
*S. cerevisiae+L. bulgaricus*	0	89.62 ± 0.12^a^	10.23 ± 0.12^a^	1.62 ± 0.07^a^	2.99 ± 0.10^a^	7.01 ± 0.02^a^	75.63 ± 0.21^a^	12.75 ± 0.17^a^
	5	47.92 ± 0.10^b^	52.08 ± 0.10^b^	1.16 ± 0.06^b^	2.87 ± 0.07^a^	7.03 ± 0.05^a^	71.14 ± 0.14^b^	18.80 ± 0.05^b^
	10	47.63 ± 0.52^b^	52.37 ± 0.52^b^	1.31 ± 0.02^c^	2.93 ± 0.14^a^	6.88 ± 0.07^b^	70.44 ± 0.54^b^	18.43 ± 0.34^b^

## Data Availability

The experimental data used to support the findings of this study are included in the article.
